# Potential of Circulating miRNAs as Molecular Markers in Mood Disorders and Associated Suicidal Behavior

**DOI:** 10.3390/ijms24054664

**Published:** 2023-02-28

**Authors:** Bhaskar Roy, Shinichiro Ochi, Yogesh Dwivedi

**Affiliations:** 1 Department of Psychiatry and Behavioral Neurobiology, University of Alabama at Birmingham, Birmingham, AL 35294, USA; 2 Department of Neuropsychiatry, Molecules and Function, Ehime University Graduate School of Medicine, Shitsukawa, Toon 791-0295, Ehime, Japan

**Keywords:** biomarker, miRNA, depression, bipolar disorder, suicidal behavior

## Abstract

Mood disorders are the most prevalent psychiatric disorders associated with significant disability, morbidity, and mortality. The risk of suicide is associated with severe or mixed depressive episodes in patients with mood disorders. However, the risk of suicide increases with the severity of depressive episodes and is often presented with higher incidences in bipolar disorder (BD) patients than in patients with major depression (MDD). Biomarker study in neuropsychiatric disorders is critical for developing better treatment plans by facilitating more accurate diagnosis. At the same time, biomarker discovery also provides more objectivity to develop state-of-the-art personalized medicine with increased accuracy through clinical interventions. Recently, colinear changes in miRNA expression between brain and systemic circulation have added great interest in examining their potential as molecular markers in mental disorders, including MDD, BD, and suicidality. A present understanding of circulating miRNAs in body fluids implicates their role in managing neuropsychiatric conditions. Most notably, their use as prognostic and diagnostic markers and their potential role in treatment response have significantly advanced our knowledge base. The present review discusses circulatory miRNAs and their underlying possibilities to be used as a screening tool for assessing major psychiatric conditions, including MDD, BD, and suicidal behavior.

## 1. Introduction

Major depressive disorder (MDD) is one of the most prevalent psychiatric disorders, affecting about 15–17% of the population in the United States, and is associated with significant disability, morbidity, and mortality. Bipolar I disorder, characterized by recurrent manic and depressive episodes, is estimated to have a 12-month prevalence of 0.6% to 2.8% [1,2]. Bipolar II disorder, characterized by a history of a hypomanic episode without mania, along with depressive episodes, has an estimated 12-month prevalence of 0.8% [1]. Patients with MDD and BD, especially those who are untreated, are at high risk for suicide. Most people with MDD or BD do not attempt or die by suicide, but both these disorders are linked to a greater risk of suicide. The estimated lifetime risk of suicide among MDD and BD patients is around 20% [3,4]; however, individuals with bipolar disorder are up to 20 times more likely to attempt suicide [5]. One of the challenges in the mental health field is the lack of consensus on the diagnosis. Also, a considerable number of mood disorder patients do not respond to the available medications. The primary reason is an incomplete understanding of the processes underlying these disorders, along with the limited availability of biologically based guidance for clinicians to effectively predict the development of mood disorders in patients at risk and prescribe medications that can effectively treat these patients.

Changes in miRNA expression in plasma, serum, and cerebrospinal fluid (CSF) are an early indication of pathological changes in the brain. Unlike many other disciplines of medicine, the potential use of miRNAs as peripheral biomarkers is less appreciated in the psychopathological evaluations of mental disorders [6]. Lately, a significant number of clinical studies have shown coherent changes in miRNA expression between circulating blood and the brains of patients with psychiatric illnesses [7,8,9]. Over the past decade, increasing knowledge has helped understand miRNAs’ role in controlling gene expression by targeting RNA transcripts and diminishing their post-transcriptional output [10,11]. The altered genetic turnover can cause collateral changes in key cellular pathways [12]. Often these changes are associated with molecular pathologies related to disease development and progression [13]. Protracted miRNA expression modulation causes rippling effects ranging from gene expression alteration to a shift in cellular and behavioral phenotypes [14,15]. Similarly, the role of miRNAs in the brain is associated with the repatterning of gene expression changes and is the cause of improper neuronal functioning [16]. Empirically, several human postmortem brain studies have shown a divergent role of miRNAs in modulating gene functionalities in subjects with affective disorders and those having suicidal behavior or who died by suicide [17]. Based on these studies, the ability of miRNAs to change gene function has been a focus of the recent investigation to understand their role as an epigenetic modifier in the pathogenicity of mood disorders and vulnerability to developing suicidal behavior [18,19].

With remarkable breakthroughs in disease diagnosis and treatment development, biomarker discovery has become increasingly popular in the early detection of diseases and the timely limiting of their progression through drug discoveries [20]. Colinear changes in miRNA expression between the brain and systemic circulation have added great interest to examining their potential as molecular markers in mental disorders, including MDD, BD, and, most importantly, suicide [6]. There are multifold advantages of using miRNAs as biomarkers in psychopathology. For example, in addition to the correlative changes between brain and blood, the relative ease in assaying miRNAs in systemic circulation (including plasma, serum, saliva, and CSF) potentially positions them as prominent diagnostic and prognostic markers [21]. There is another salient feature of miRNAs that makes them excellent biomarkers. miRNAs are secreted in peripheral circulation in an enclosed vesicular structure or extracellular vesicles [22]. Vesicle-bound miRNAs often carry the molecular signature associated with the tissue of origin. In other words, miRNAs in free-floating extracellular vesicles are postmarked by their source tissue. This gives another dimension to the miRNA-based biomarker discovery. Besides extravesicular cargo, miRNAs in circulation can also be traced that are conjugated with proteins such as argonaute 2 (Ago2) and high-density lipoprotein (HDL). Most of the time, the role of these conjugated proteins is to provide stability and protection to miRNAs from nuclease activity [23]. Collectively, both classes constitute the family of circulating miRNAs with the potential to be used as peripheral biomarkers in neuropsychiatric disorders, such as MDD, BD, and suicidal behavior. 

The present review intends to spotlight the studies that have drawn significant attention to our understanding of the role of circulating miRNAs as potential biomarkers in three major neuropsychiatric conditions: MDD, BD, and associated suicidal behavior. Some of these miRNAs have the ability to be used as predictive biomarkers. In contrast, some have the potential to be categorized as treatment response biomarkers and are likely to be associated with risk prediction. This review also examined the similarities and differences of dysregulated circulating miRNAs between MDD and BD. Altogether, we have made a comprehensive overview of the literature to summarize the role of miRNAs in circulation and their potential value in diagnosing MDD, BD, and suicidal behavior based on their peripheral screening.

## 2. Materials and Methods

The primary criteria used to search the circulating miRNA-associated reports were studies highlighting the role of circulating miRNA as peripheral biomarkers in MDD, BD and suicidality. With the help of PubMed database the search was performed to primarily include original articles for the last ten years using various keywords to increase the chance of article retrieval, falling under the criteria mentioned previously. The following keywords were used for retrieval: “circulating microRNAs” AND “Major depressive disorder” OR “MDD” OR “Major depression” AND “Bipolar disorder” OR “BD” AND “Suicide” OR “Suicidality” AND “Biomarker.”

## 3. Overview of miRNA Biogenesis 

Mammalian miRNA biogenesis is a programmed cellular event and starts with their transcription in the nucleus with the help of RNA polymerase II/IIII (RNA Pol II/Pol III) [24]. The transcription often starts in the genomic loci, where miRNA coding units are engraved alongside mRNA coding units. The immediate product of transcription is the primary transcript (pri-miRNA) with a relatively larger size of ~1kilobase (kb). The pri-miRNAs structure consists of a stem terminal loop flanking overhang on both 5′ and 3′ ends. In the nucleus, the pri-miRNA triggers a self-cleaving process with the help of ribonuclease III enzyme Drosha and ancillary protein factor Dgcr8. Together, they form a microprocessor complex to crop off the overhangs on both sides of the pri-miRNA. The activity helps to release a shorter hairpin 65 nucleotides long and is called precursor miRNA or pre-miRNA [25,26]. This marks their exit from the nuclear environment toward cytosol with the help of active transportation by the Exportin 5 nuclear membrane complex. The release of pre-miRNA into the cytoplasm facilitates further enzymatic processing by the RNase III Dicer. The cleavage by Dicer finally generates mature miRNAs and makes them accessible to the argonaut 2 family protein. Finally, mature miRNAs are incorporated into the RNA-induced silencing complex (RISC), which helps them to regulate the fate of target gene expression by pairing primarily to the 3′untranslated region (3′UTR) of protein-coding mRNAs [27]. Once available in mature form, miRNAs participate in the post-transcriptional regulation of target transcripts by either depleting their endogenous level with the help of RNA RISC or by modulating translation machinery recruited on the coding transcript. MiRNAs function as master regulators of gene expression at the post-transcriptional level either by modulating the mRNA translation or transcript degradation by targeting the 3′UTR region [28]. 

## 4. Outlining the Pathogenic Role of miRNA in Neuropsychiatric Brain

Being a complex structure, the brain constantly faces countless challenges to maintain its homeostatic stability [13]. Most of the time, these challenges are associated with aversive environmental stimuli and carry the potential to discourse normal brain functioning via epigenetic factors such as miRNAs [29,30]. Aberrant gene expression regulation is often correlated with abnormal brain functioning and, to a large extent, targeted by miRNA molecules [29,31]. miRNAs act as molecular switches to flip the sides of gene regulatory workflow from a normal to a disease state [18]. Most prevalent neuropsychiatric illnesses, including MDD, BD, and suicidal behavior, have often shown discernable changes in miRNA expression [17,32]. Most of the time, the changes result from converging environmental challenges considered to be the precipitating factors associated with these mental disorders [33]. Over the years, an increasing number of studies have pointed out the neuropathogenic roles of miRNAs in MDD, which act in various capacities spanning from mRNA transcript sequestration to the competitive endogenous inhibition of coding genes [34,35]. However, most of the activities are associated with either anomalous gene expression regulation at the candidate level or the desynchronization of a large-scale gene regulatory network at the genomic level [35]. Some of the notable reports of miRNA changes in the MDD brain include the anterior cingulate cortex [ACC], dorsolateral prefrontal cortex [BA] 9, other prefrontal cortical areas (BA10, BA44, BA46), and the locus coeruleus [LC]), which are known for their roles in mood disorders [17,36,37]. Studies from our lab have provided an advanced understanding on how miRNA, being a small epigenetic regulator, can be associated with underlying molecular changes in the MDD brain [7,38]. Our group, for the first time, explored miRNA expression changes in the brain of suicide subjects diagnosed with MDD. A total of 21 miRNAs were found significantly downregulated compared to healthy control subjects [39]. Surprisingly, the overall change in miRNA expression had a decreasing trend in MDD-suicide subjects. In the same report, several overlapping target genes were found among 21 dysregulated miRNAs. Later, we studied synaptosomal miRNAs from BA10 of the MDD brain [40]. The expression of miR-508-3p and miR-152-3p was significantly downregulated in the MDD brain compared to control subjects. However, miR-508-3p expression in suicide subjects was significantly lower than in non-suicide MDD subjects. In another study, we showed the downregulation of miR-124-3p expression in BA46 of MDD subjects [7]. For the same miRNA, parallel expression changes were found in the serum of antidepressant-free MDD patients. Moreover, the expression of miR-124-3p was suppressed by fluoxetine treatment [7]. Recently, we also explored the miRNA-related changes in LC of MDD suicide subjects on a semi-high throughput expression platform [17]. A total of ten upregulated and three downregulated miRNAs were detected in MDD subjects. Based on target gene prediction using the upregulated miRNAs, we narrowed our study to genes with a robust neuropsychiatric relationship (RELN, GSK-3β, MAOA, CHRM1, PLCB1, and GRIK1). Of those, reduced expression levels were found for RELN, GSK-3β, and MAOA genes [17].

## 5. miRNAs as Clinical Biomarkers in MDD

Recently, a sizeable number of studies have been published to highlight the true potential of miRNAs to be used as biomarkers in circulation for MDD, BD, and suicidal behavior. Although it is rather impossible to track down peripheral changes in patients who died by suicide, a few studies have shown altered miRNA responses in circulation to be associated with an increased risk of suicide. In the following section, we have described some key findings published over the past few years that have contributed substantially to understanding circulating miRNAs’ role as molecular biomarkers in MDD diagnosis (Table 1).

Maffioletti et al. [41] tested the whole blood expression of 1733 miRNAs using microarrays in patients with MDD (n = 20) as compared to those with BD (n = 20) and healthy controls (n = 20). Ten miRNAs were altered in MDD compared to controls. More precisely, the authors noted MDD-specific changes in let-7a-5p, let-7d-5p, let-7f-5p, miR-24-3p, and miR-425-3p. The authors also used bioinformatic analysis to determine signaling pathways targeted by these miRNAs and found that Wnt and mTOR signaling were the main targets. In a separate study, an Affymetrix expression array was used to determine miRNA expression changes in blood mononuclear cells (PBMC) [42]. The array identified 26 miRNAs with significant changes in expression in MDD patients. The data were further validated in a larger cohort of 81 MDD patients and 46 healthy controls. The qPCR-based outcome confirmed the upregulated expression of five miRNAs (miRNA-26b, miRNA-1972, miRNA-4485, miRNA-4498, and miRNA-4743). Receiver operating characteristic (ROC) curve analysis determined a confidence level of 0.636 (95% confidence interval (CI): 0.58e0.90) for those five miRNAs. The authors also used these miRNAs in target gene prediction and their functional annotation. The functional analysis identified pathways associated with the nervous system and brain functions. Collectively, the report supports the principle of using blood-based miRNA expression changes in MDD diagnosis [42]. 

In another study, Camkurt et al. [43] investigated miRNA expression changes in MDD patients using plasma drawn from the venous blood of 50 depressed patients and 41 healthy controls. qPCR-based expression analysis showed significant changes in four miRNAs when MDD patients were compared with healthy controls. The three in the upregulated group were miR-451a, miR-17-5p, miR-223-3p, and the downregulated one was miR-320a. According to the authors, miR-451a could be a candidate biomarker for depression. 

Our lab showed the expression changes of miR-124-3p in the postmortem brain samples from MDD patients [7]. Following the same trend, a significant ~3.5-fold increased expression in miR-124-3p was noted in the serum of MDD patients free from any antidepressant treatment. Our study implicated for the first time the role of stress-induced miR-124-3p as a potential biomarker in MDD pathogenesis. The role of miR-124 was further supported by another peripheral study in MDD patients [44]. This study investigated the expression changes in miR-124 in the peripheral blood mononuclear cells (PBMCs). The expression changes were examined in 32 pre- and post-treatment MDD patients and 30 healthy controls. The results showed significantly higher expression of miR-124 in MDD patients, and the sensitivity was determined as 83.33% with ROC analysis. Interestingly, the specificity for MDD patients was 66.67%, clearly distinguishing them from healthy controls. More interestingly, the level of miR-124 expression was significantly downregulated in MDD patients after eight weeks of antidepressant treatment. These results further consolidate the potential use of miR-124 as a blood-based biomarker for MDD diagnosis and treatment response [44]. 

In another study, the authors were interested in determining miRNA changes in the serum of perioperative patients who had developed depression [45]. They primarily examined if specific miR-221-3p could be used as a biomarker for depressed mood in perioperative patients. In their study plan, two sets of perioperative patients were included having either mild depressive mood or moderate to severe depressive mood. Serum-based qPCR data suggested a greater than 2-fold significant expression upregulation of miR-221-3p in both the depressed groups compared to the control group. Correlation analysis between serum-based miR-221-3p expression and depressed mood score established a positive relationship, suggesting that the elevated expression of miR-221-3p can serve as a biomarker for depressive mood in perioperative patients. The authors further pointed out the ability of miR-221-3p to induce the expression of interferon (IFN)-α in astrocytes by targeting IRF2. The miR-221-3p could be mechanistically connected with anti-inflammatory pathways via the IFN- α signaling cascade [45]. 

Besides blood, CSF has also been used to study the level of miRNA changes in depression. A study by Wan et al. [46] analyzed the expression of 179 miRNAs from a PCR panel using CSF from MDD patients. The PCR data detected significant changes in CSF miRNAs; eleven were upregulated, and five were downregulated. The CSF results were further verified in the same patient serum samples, and the results found similar changes in three miRNAs (out of eleven miRNAs) from the upregulated group but only one miRNA (out of five miRNAs) from the downregulated group. All four miRNAs (upregulated: miR-221-3p, miR-34a-5p, let-7d-3p, and downregulated: miR-451a) were further validated in a larger cohort of thirty-two MDD patients. The ROC analysis responded with a higher degree of sensitivity and specificity (the majority were above the 80% scale) for all four miRNAs in MDD diagnosis. A similar study showed a strong anti-correlation of CSF miR-16 with MDD [47]. This study found a significantly lower level of miR-16 expression in the CSF of 36 drug-free patients and a significantly lower amount of serotonin in the same CSF samples. However, testing the miR-16 expression in the blood sample of the same MDD patients did not identify any significant changes when compared with healthy control subjects. For the first time, the study showed a direct relationship between miR-16 and serotonin levels using CSF from MDD patients. In a separate report by Sun et al. [48], changes in two miRNAs (miR-34b–5p and miR-34c–5p) based on peripheral blood leukocytes were strongly associated with MDD. In addition, the study also determined an increased risk of suicide and cognitive decline associated with miR-34b–5p and miR-34c–5p expression levels. The study included 32 MDD patients with a matching number of healthy controls and analyzed the expression of miR-369-3p, miR- 34b-5p, miR-34c–5p, miR-381, and miR-107 in blood leukocytes. The study concluded that defective Notch signaling functions could potentially be associated with miR-34b-5p and miR-34c-5p out of the five miRNAs studied in MDD patients. 

Instead of showing the potential of known and characterized miRNAs as biomarkers in MDD diagnosis, a study by Zhao et al. [83], for the first time, reported a novel miRNA, pmiR-chr11, in MDD patient blood. The study was designed to explore the miRNome-wide changes in a small exploratory cohort of 10 MDD and 10 control subjects. The significant findings from the exploratory cohort were further validated in a larger cohort size of 72 MDD and 75 control cases. Among 10 significantly altered miRNAs from the exploratory cohort, only pmiR-chr11 was found to be significantly upregulated in MDD patients as compared to controls. Prediction analysis identified Bromodomain and PHD finger-containing protein 1 (BRPF1) as potential target genes strongly associated with hippocampal volume. In vitro validation experiments further supported BRPF1 as the direct target of novel pmiR-chr11. Taking the lead from blood-based miRNA changes, the authors suggested a possible role of BRPF1 in hippocampal neurogenesis, which could be affected by the increased expression of pmiR-chr11 in the MDD brain.

Early life adversities in the form of childhood maltreatment (CM) could be a major risk factor for developing depression during adulthood. He et al. [49] found an association of miR-9 with CM in the peripheral blood of 40 untreated MDD patients and 34 healthy controls. The study also found miR-9 to play a potential role in making changes in the functions of the prefrontal limbic regions of MDD patients examined through resting-state fMRI scans, which could be associated with early CM experience. 

A recent study investigating the miRNA expression changes in the plasma samples of MDD patients (84 MDD cases) found 11 miRNAs to be upregulated from miRNA sequencing data [50]. After adjusting the odds and considering the expression detection limit following qPCR, only two miRNAs (let-7e-5p and miR-125a-5p) were found to be potentially associated with MDD. Both let-7e-5p and miR-125a-5p were upregulated with moderate diagnostic sensitivity and specificity. From these results, let-7e-5p and miR-125a-5p could be added to the potential list of biomarkers in MDD diagnosis.

Understanding the role of inflammation in MDD has been a long-standing interest; however, only a few studies have attempted to discover the association of peripheral blood miRNAs with depression and its severity related to inflammatory signaling. A study published by Hung et al. [51] analyzed the expression of let-7e, miR-21-5p miR-145, miR-223, miR-146a, and miR-155 in the PBMC of 84 MDD patients before and after antidepressant treatment. The PBMC data showed lower levels of let-7e, miR-146a, and miR-155 in MDD patients than in healthy controls and were significantly higher in patients after four weeks of antidepressant treatment. On the contrary, miR-146a and miR-155 in PBMC were lower in MDD patients and increased after receiving antidepressant treatment. Interestingly, the depression severity was found to be inversely correlated with let-7e and miR-146a expression, whereas a direct correlation was found for miR-155. These miRNAs are part of toll-like receptor signaling pathways directed toward the TLR4 system. Therefore, the changes in these TLR4-regulating miRNAs in MDD patients and their association with depression severity and their responsiveness to AD treatment could be used as biomarkers in diagnosis and treatment response.

An intriguing part of devising MDD treatment is understanding the periodic relapse common in patients formerly treated with antidepressants. Analyzing miRNA expression changes in the peripheral circulation of MDD-relapsed cases could be valuable in developing potential biomarkers for recurring MDD. One such study by Li et al. [52] examined miRNA expression changes in the serum samples of 63 relapsed and 154 non-relapsed MDD patients. A lower level of expression was noted for four miRNAs (miR-199b- 5p, miR-143-3p, miR-200a-3p, and miR-215-5p). The low expression levels of these four miRNAs were also associated with a lower risk of future relapse. The predicted targets of these four miRNAs and their functional clustering identified several neurobiological functions and pathways that included neurogenesis, response to cytokine, neurotrophin signaling, vascular endothelial growth factor signaling, relaxin signaling, and cellular senescence pathways.

Interestingly, miRNA can be used as a biomarker for predicting the disease development trajectory, as reported in a nested case-control study by Roumans et al. [53]. In this study, 104 cases were enrolled as MDD-free and 52 of them developed MDD in the course of the following five years. The remaining 52 participants did not develop any MDD and were treated as a control to compare the miRNA findings. The expression levels of five miRNAs (miR-17-5p, miR-134-5p, miR-144-5p, let-7b-5p, and let-7c-5p) were analyzed from plasma at the baseline. After adjustments of all odds, the expression level of let-7b-5p was significantly lower at the baseline. Interestingly, the level of let-7b-5p remained significantly lower and was negatively associated with developing MDD. This is one of the notable studies showing that the future risk of developing MDD can be related to peripheral miRNA expression and could be used as a biomarker for MDD risk prediction.

## 6. miRNAs as Biomarkers of Treatment Response in MDD Patients

A study published by Gururajan et al. [54] showed miRNAs’ relationship with two effective therapies in MDD patients who had an inadequate response to two consecutive antidepressant treatment regimens of different pharmacological classes. The study was conducted to identify changes in peripheral miRNA expression in 40 MDD patients who were clinically diagnosed with TRD and received electroconvulsive therapy (ECT) and ketamine infusion. In the ECT group, 24 patients were tested for genome-wide miRNA expression in blood. The same procedure was followed in the ketamine treatment group, where 16 patients were screened for miRNA changes. Expression data were compared between cases and controls at baseline and after treatments. Although decreased levels were noted for let-7b and let-7c at baseline and after ECT, no significant miRNA changes were noted for ketamine treatments as determined by qPCR. This study concluded that the baseline expression of miRNAs could not be a good predictor of treatment response. However, the trending changes in let-7b and let-7c expression could potentially be used as biomarkers for TRD. 

Another study examined changes in miR-134 in 100 MDD patients treated with antidepressants [55]. At the baseline, the plasma miR-134 was significantly downregulated in the MDD group compared to the control group. After eight weeks of follow-up with AD treatment, significant expression upregulation of miR-134 was noted in partially responsive and fully responsive MDD subjects. However, a non-significant change was found in the mean plasma level of miR-134 when the non-responders were considered independently. The diagnostic accuracy of miR-134 was determined in MDD patients following ROC and was found to be 0.901 with an AUC. The sensitivity and specificity of miR-134 in diagnosing MDD were 79 and 84%, respectively. Overall, the study suggested a reduced level of plasma miR-134 in the acute phase of MDD independent of any AD treatments. Additionally, the study found an increasing level of plasma miR-134 with the symptomatic improvement of MDD following eight weeks of AD treatment. However, it has also been noted that patients with minimal plasma miR-134 may not respond well to conventional AD treatments. Altogether, responses in miR-134 can be effectively used as a state-dependent biomarker in MDD prognosis. 

Recently, circulating miR-144-3p has been identified as a molecular marker to predict depression severity and treatment response biomarker to ketamine [56]. The study determined a sex-independent higher level of miR-144-3p expression at the baseline of MDD and a positive correlation with depression severity. However, a male-specific reduction in miR-144-3p expression was determined based on a correlation with ketamine treatment response. Similar changes in miR-144-3p were noted in the mouse model of chronic social defeat stress. The reversal in expression was evident when chronically stressed mice were treated with repeated doses of imipramine or a single dose of ketamine. The study also successfully antagonized the stress-inducing effect of miR-144-3p by systematically injecting antisense miR-144-3p locked nucleic acid in mouse blood. The systemic knockdown of miR-144-3p was sufficient to reduce the depression-like phenotype in mice. 

Understanding the role of miRNAs in the mechanism of antidepressant treatment response has been a field of interest for a long time. For the first time, Bocchio-Chiavetto et al. [57] analyzed the global miRNA expression in 10 depressed subjects after ten weeks of escitalopram treatment. Genome-wide miRNA expression data suggested changes in 30 miRNAs in AD-treated MDD subjects. Among 30 analyzed miRNAs, 28 were upregulated, and the remaining 2 were downregulated. This study also noted that the potential targets of all the altered miRNAs had demonstrated functional enrichment for neuronal pathways, including neuroactive ligand–receptor interaction, axon guidance, long-term potentiation, and long-term depression. Lopez et al. [58] used next-generation sequencing in MDD patients treated with duloxetine and found the downregulation of several miRNAs that targeted Wnt and MAPK signaling. They found that six miRNAs were altered by treatment and placebo and hypothesized that they might be responsible for a common response to antidepressant treatment. After replication in patients treated with escitalopram and in human postmortem brains, their study showed that miR-146b-5p, miR-24- 3p, and miR-425-3p were downregulated by AD treatment and upregulated in the brains of patients who died by suicide. The MAPK and WNT signaling pathways were found to be strongly associated with these miRNAs. This evidence supports that the downregulation of miR-146b-5p, miR-24-3p, and miR-425-3p may be related to improved symptomatology via increased MAPK and WNT signaling. These miRNAs also seem to be consistently and significantly associated with behavioral responses to antidepressants and showed strong promise as clinical biomarkers. 

Receptors for neurotransmitters remain key targets for pharmacological manipulation and potential drug development in MDD. Glutamatergic receptor GRM4 is one such target with a role in MDD pathogenesis. Interestingly, miR-335 has been reported to play a significant role in modulating the GRM4 receptor using [59]. The authors in this study showed the significant downregulation of miR-335 in the blood samples of MDD patients compared with healthy controls. Measuring miR-335 and GRM4 in the peripheral blood samples of MDD patients treated with citalopram showed that the expression of miR-335 was upregulated. In contrast, the GRM4 level was decreased significantly in MDD patients compared to healthy controls. This was the first study to show a mechanistic relationship between a miRNA and a neurotransmitter receptor and highlighted the use of miRNAs as potential biomarkers in predicting treatment response in MDD patients.

A small study by Enatescu et al. [60] analyzed the expression of 222 miRNAs in the plasma samples of five MDD patients to identify treatment-specific miRNA changes. The results from the analysis determined differential changes in 40 miRNAs in response to AD treatment. Among 40 differentially expressed miRNAs, 23 were significantly upregulated, and 17 were significantly downregulated in MDD patients compared to control subjects. In further analysis to understand the functional contribution of the 40 dysregulated miRNAs, a prediction algorithm identified pathways related to Wnt signaling, endocytosis, axon guidance, and MAPK signaling. The authors found six key miRNAs primarily enriching four of the five above-mentioned functional pathways. A later study based on the plasma samples of MDD patients identified the responsiveness of miR-132 and miR-124 to treatment [61]. The results showed the significant enrichment of miR-132 expression in MDD patients compared to control subjects and MDD patients treated with citalopram for two months. On the other hand, changes in miR-124 expression in untreated and citalopram-treated MDD patients were significantly higher than in control subjects. The authors also examined the BDNF level in the plasma samples of both MDD groups following the immunosorbent assay and found a trending increase. Additionally, the authors determined a positive correlation between increasing miR-132 levels in plasma and depression severity based on HAMD and HAMA scales. The authors concluded that plasma-based expression changes in miR-132 could be used as a diagnostic biomarker in determining depression status in clinical settings. 

Another study analyzed ten miRNAs (miR-16, miR-30, miR-34, miR-128, miR-132, miR-134, miR-182, miR-183, miR-185, and miR-212) in the serum of MDD patients treated with either selective serotonin reuptake inhibitor (SSRI) or serotonin–norepinephrine reuptake inhibitor (SNRI) [62]. The study aimed to determine the peripheral changes in miRNAs associated with the BDNF signaling pathway. In the study, 13 patients received SSRI treatments, whereas 20 were given SNRIs. After analyzing all data sets from the two treatment groups, only miR-16 showed a significant increase in the SSRI-treated group. After adjustment and multiple corrections, no significant changes were identified for other miRNAs in both SSRI and SNRI groups. However, after four weeks of AD treatments, the Wilcoxon signed rank test identified an overall significant increase in miR-183 and miR-212 levels in MDD patients. The findings from this study are critical to understanding the role of miRNAs as predictors of AD treatment. Their differential responsive pattern under different pharmacological agents could be vital in selecting the biomarker panel for proper clinical management. 

## 7. Circulating miRNAs in BD

This section presents a series of studies showing how circulating miRNAs could be associated with BD. A list of miRNAs related to BD is shown in Table 1. In the plasma of 66 BD patients who were treated with lithium and 66 control subjects, Tekdemir et al. [69] investigated plasma miRNAs and found a significant increase in miR-132, miR-134, miR-152, miR-607, miR-633, and miR-652, and a significant decrease in miR-15b and miR-155 levels in BD patients. They suggested that an increase in miR-134-5p, miR-652-3p, and a decrease in miR-15b and miR-155-5p were associated with the risk of BD. They found that miR-155-5p was explicitly associated with the disease burden and severity. Fatty acid biosynthesis and metabolism, viral carcinogenesis, the EBV infection, and extracellular matrix and adhesion pathways were highlighted as target pathways. In whole peripheral blood from 56 (25 females and 31 males) BD-I patients and 52 (26 females and 26 males) control subjects, Tekin et al. [72] demonstrated a significant increase in miR-376a-3p, miR-3680-5p, miR-4253-5p, and miR-4482-3p levels and a significant decrease in miR-145-5p level in BD-I patients. They also revealed that miR-145-5p targeted the dopamine decarboxylase (DDC) gene, which could serve as a biomarker for BD-I.

Interestingly, Lee et al. [71] explored the possibility of serum miRNAs as specific biomarkers for BD-II. Using next-generation sequencing, they identified six miRNAs to be differentially regulated that can differentiate BD-II patients from controls. These candidate miRNAs were confirmed with real-time PCR in a cohort of 79 BD-II and 95 controls. A diagnostic model was built based on these candidate miRNAs and then tested on an individual testing group (BD-II: n = 20, controls: n = 20). They found that the serum levels of miR-7-5p, miR-23b-3p, miR-142-3p, miR-221-5p, and miR-370-3p were significantly increased, whereas miR-145-5p had no significant difference in BD-II patients compared with controls. Support vector machine measurements revealed that a combination of the significant miRNAs reached good diagnostic accuracy (AUC: 0.907). In a follow-up study, the same group investigated the correlation between miR-7-5p, miR-142-3p, miR-221-5p, and miR-370-3p with BDNF levels using the serum of 98 (65 females and 33 males) BD-II patients [77]. They revealed that miR7-5p, miR221-5p, and miR370-3p were significantly correlated with the plasma BDNF levels, and miR142-3p was significantly correlated with durations of illness. They also analyzed a correlation of these miRNAs with BDNF Val66Met polymorphism and found that miR-221-5p and miR-370-3p were significantly correlated with BDNF in only the Val/Met genotype and miR-7-5p in all three genotypes.

Another group of investigators examined miRNAs in BD patients using plasma [67]. In 69 BD patients and 41 controls, they reported a significant increase in miR-185-5p, miR-25-3p, miR-92a-3p, miR-376b-3p, and let-7i-5p and a significant decrease in miR-484, miR-652-3p, miR-142-3p, miR-30b-5p, miR-126-3p, miR-15a-5p, miR-126-5p, and miR-301a-3p levels in BD patients. From Benjamini–Hochberg correction, they revealed that miR-185–5p was significantly increased, and miR-484, miR-652–3p, and miR-142–3p were significantly decreased and suggested that these four microRNAs could be used as biomarkers with a specificity of 75.0% and a sensitivity of 87.1%. In a similar fashion, Fries et al. [68] examined miRNAs in the plasma of 20 BD-I patients and 21 controls. They revealed that a set of 33 microRNAs were significantly different in the BD group compared to the control group and were associated with netrin and endothelin signaling, 5HT2 receptor-mediated signaling, β1 and β2 adrenergic receptor signaling, and androgen receptor signaling. Most of these miRNAs differed from what was previously reported by Ceylan et al. [67]. More recently, using a comprehensive literature search and data mining approach, a report suggested that miR-106b, miR-125a, miR-142, miR-221, and miR-652 can be used as circulating miRNAs for diagnosing BD [84]. 

A few studies have independently examined mania and euthymia’s impact on miRNA expression. To determine if specific miRNAs are associated with psychotic manic episodes in BD patients, in plasma samples from a group of 15 BD patients and 9 control subjects, Tabano et al. [70] reported a significant increase in miR-150-5p, miR-25-3p, miR-451a, and miR-144-3p, and a significant decrease in miR-363-3p, miR-4454, miR-7975, miR-873-3p, miR-548, miR-598-3p, miR-4443, miR-551a, and miR-6721-5p, suggesting that miRNA changes may differ in manic patients within the BD group. Functionally, the increased miRNAs were associated with metabolic regulation and the decreased ones with neurogenesis and neurodevelopment. In a similar line of investigation, another study examined miRNAs in plasma from 58 BD-I patients with manic and euthymic episodes (19 with mania and 39 with euthymia) and 51 controls [74]. It was found that compared to controls there was a significant increase in miR-9-5p, miR-29a-3p, miR-106a-5p, miR-106b-5p, miR-107, miR-125a-3p, and miR-125b-5p in BD patients with manic episodes, and a significant increase in miR-29a-3p, miR-106b-5p, miR-107, and miR-125a-3p in BD patients with euthymic episodes. They also showed that miR-106a-5p and miR-107 in BD with manic episodes were more significantly increased than in the euthymic episodes. This study clearly demonstrates state-specific miRNAs in BD patients. 

## 8. Effect of Medications on miRNAs in BD Patients

Interesting results were noted when miRNAs were examined in BD patients with and without treatment. An examination of plasma miRNAs from 21 (7 females and 14 males) BD patients with manic episodes who did not receive any medication and 21 (7 females and 14 males) healthy controls found that the level of miR-134 was significantly decreased in BD patients [85]. Interestingly, following medication, miR-134 levels gradually increased from baseline. After four weeks of medication, miR-134 levels were significantly increased from baseline, but compared to controls miR-134 levels in BD patients were still considerably lower. Another study investigated changes in miRNAs before and 12 weeks after asenapine or risperidone treatment from blood samples of 10 BD-I patients with manic episodes [75]. The investigators reported a significant increase in miR-15a-5p, miR-17-3p, miR-17-5p, miR-18a-5p, miR-19b-3p, miR-20a-5p, miR-27a-3p, miR-30b-5p, miR-106a-5p, miR-106b-5p, miR-145-5p, miR-148b-3p, miR-210-3p, and miR-339-5p, and a significant decrease in miR-92b-5p and miR-1343-5p in the asenapine group from baseline. There was a significant decrease in miR-146b-5p, miR-664b-5p, and miR-6778-5p in the risperidone group.

## 9. Parallel miRNA Studies Comparing BD and MDD 

Examining the differences in miRNA expression between MDD and BD is critical. A few studies have attempted to explore this differentiation by paralleling examing miRNA expression in circulatory blood samples from BD and MDD patients. A study investigated miRNAs in blood samples from 20 BD patients, 20 MDD patients, and 20 controls [41]. In this study, compared to controls, a significant increase in miR-21-3p, miR-29c-5p, miR-30d-5p, miR-140-3p, miR-330-3p, miR-330-5p, miR-345-5p, miR-378a-5p, miR-720-5p, miR-1973-5p, miR-3158-3p, and miR-4521-5p was observed in BD patients. In addition, a significant decrease in miR-1915-5p, miR-1972-5p, miR-4440-5p, and miR-4793-3p was also found in BD patients. Interestingly, miR29c-5p, miR-330-3p, and miR-345-5p were increased in both MDD and BD compared with controls. The study also found a more significant upregulation of miR-21-3p, miR-30d-5p, miR140-3p, miR-330-5p, and miR378a-5p in BD patients than MDD and controls. 

miRNA expression levels were also examined in PBMC from 63 (26 females and 37 males) BD patients, 42 (18 females and 24 males) MDD patients, and 57 (26 females and 31 males) controls [73]. The study revealed a significant increase in miR-499-5p in BD patients but not MDD patients compared to controls. This miRNA was found to regulate calcium voltage-gated channel auxiliary subunit beta 2 (CACNB2) in the mouse hippocampus, which has been implicated in BD. 

In addition to comparing miRNAs between MDD and BD, one study examined specific miR-134 in BD, MDD, and schizophrenia patients [55]. In a cohort of 50 BD, 100 MDD, 50 schizophrenia, and 100 control subjects, the study found that plasma miR-134 was significantly downregulated in MDD patients and plasma miR-134 levels could effectively distinguish MDD from controls with 79% sensitivity and 84% specificity, while distinguishing MDD from controls, BD, and schizophrenia subjects with 79% sensitivity and 76.5% specificity.

A relatively small study in the plasma of seven BD, seven MDDD, and six control subjects found a more significantly decreased level of miR-19b-3p in BD than in MDD and control groups [76]. The study also found that the expression of miR-19b-3p in BD was significantly associated with early life stress. Interestingly, while examining plasma samples from 26 BD patients, 84 MDD patients, and 74 controls, Let-7e-5p and miR-125a-5p showed a significant increase in both BD patients and MDD patients compared with controls, these two miRNAs were not different between BD and MDD patients [50]. Interestingly, an independent study showed that miR-15b, miR-132, and miR-652 were significantly upregulated in the blood samples of high-risk mood disorder patients when examined in 34 high genetic risk mood disorder patients and 46 control subjects [78].

## 10. Circulating miRNAs in Suicidality

There is an overarching need for future miRNA biomarker discovery that can effectively articulate the risk of suicidality, including suicidal ideation (SI) and recovery from suicidal intent. As shown in Table 1, circulating miRNA in suicidality is a less studied area. A genome-wide miRNA expression profiling study determined changes in miRNA levels from 42 inpatients admitted for strong suicidal ideation [79]. The expression analysis was primarily focused on understanding the miRNA expression changes in the plasma of 42 SI patients who later recovered. The SI patients showed the decreased expression of four miRNAs (miR-424-5p, miR-378i, miR-6724-5p, and miR-10b-5p) after their recovery from SI. The authors found a close association of these miRNAs with key brain functions involving the MAPK, ErbB, AMPK, Ras, p53, and PI3K-Ak pathways. These pathways have previously been shown to be related to depression and suicidality. This exciting finding could unfold the potential of circulating miRNAs to be used as markers for the symptomatic remission of suicidal intent in the recovery phase. 

A few recent reviews compiled studies and speculated that miRNAs could be associated with suicidal behavior [6,86]. For example, an algorithm search through the miRNA database identified miR-27b-3p, miR-124-3p, miR-129-5p, miR-381-3p, miR-3135b, miR-4516, and miR-4286 that were most related to suicide [80]. A clinical study investigated miRNAs in the PBMC of 12 MDD patients with severe suicidal ideation and 12 controls [81]. The study reported that miR-19a-3p was significantly associated with suicidal behavior. This miRNA was found to regulate TNF-α, a proinflammatory gene implicated in mood disorders and suicide. To examine if miRNAs could be used as predictors of the treatment-worsening of suicidal ideation (TWSI), a recent study investigated miRNAs in the whole blood of 237 MDD patients (112 with duloxetine and 125 with placebo) [82]. A total of 11 patients with duloxetine showed TWSI, and miR-3688 and miR-5695 were significantly associated with the worsening of suicidal ideation. 

As mentioned above, a majority of the studies on suicidal patients have been conducted in the context of MDD. Of miRNAs that are studied in suicidal patients, miR-124-3p and miR-129-5p are associated with MDD [7,60,80], and miR-3135b and miR-4516 are associated with BD [68,80]. However, no previous study reported common suicide-associated miRNAs in both disorders. It will be important to dissect miRNAs associated with suicidal behavior that are common to both disorders. Also, distinguishing miRNAs that are associated with suicidality in BD patients vs. those in MDD patients will also provide a clear distinction of disease-specific suicidality-related miRNAs, given that BD patients have a higher potential for suicide than patients with MDD [87,88].

## 11. Molecular Profiling of Genes Targeted by miRNAs Derived from MDD and BD Patients

**Figure 1:**
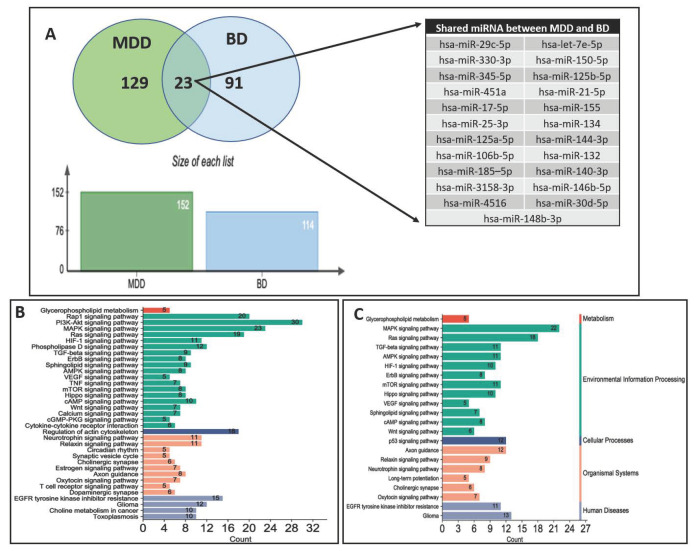
(**A**) Venn diagram showing the comparison of exclusive and shared miRNAs between MDD and BD groups. Bar plot showing total number of miRNAs in MDD (152) and BP (114) groups and a list of 23 shared miRNAs between MDD and BD are also depicted; (**B**) Bar plot showing pathway clustering based on the number of genes targeted by 129 miRNAs associated with MDD; (**C**) Bar plot showing pathway clustering based on the number of genes targeted by 91 miRNAs associated with BD.

**Figure 2:**
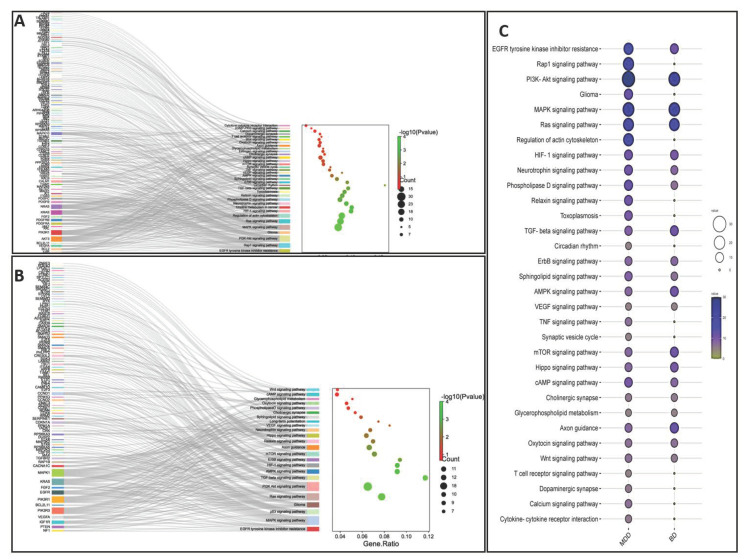
(**A**) Sankey plot showing biological pathways connected with the genes targeted by 129 MDD-associated miRNAs; (**B**) Sankey plot showing biological pathways connected with the genes targeted by 91 BD-associated miRNAs; (**C**) Comparative similarities and differences in biological pathways targeted by miRNAs selectively associated with MDD and BD.

**Figure 3:**
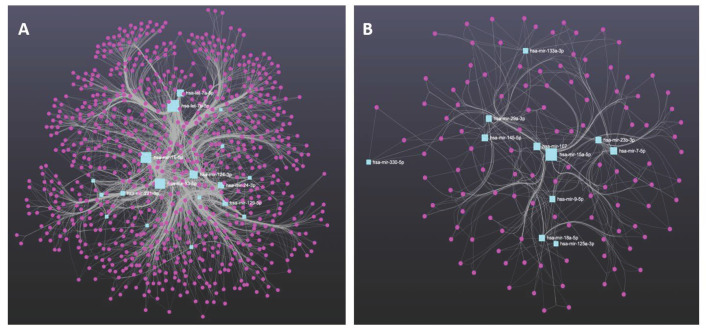
(**A**) Prediction-based miRNA-target association network using MDD-enriched miRNAs collected from the literature; (**B**) Prediction-based miRNA-target association network using BD enriched miRNAs collected from the literature.

Pathophysiological changes in MDD and BD could result from genes that lose their harmonized expression patterns due to regulatory influence by miRNAs. Based on the literature discussed in this review, we compared the list of circulatory miRNAs found to be altered in MDD and BD patients. We determined that 129 miRNAs were uniquely associated with the MDD group, 91 miRNAs were uniquely associated with the BD group, and 23 miRNAs were shared between MDD and BD groups (Figure 1A). The chromosomal coordinates, full sequences, and their current miRBase ID of the two sets of miRNAs uniquely associated with MDD and BD are provided in Table 2. We further used the unique miRNA sets from the MDD and BD groups to predict their targets independently. All predicted targets were filtered based on the brain enrichment database and independently used in functional analysis to determine the biological pathways following the Kyoto Encyclopedia of Genes and Genomes (KEGG) database. In Figure 1B,C, we have shown the individual pathways targeted by miRNA sets for MDD and BD, respectively. The pathway lists were modified to incorporate the key pathways enriched for neurobiological functions and neuropsychiatric disorders. Each bar in the bar diagram (Figure 1B,C) also represents the number of miRNA-targeted genes associated with individual pathways. In the figures, the bar plots are further classified in four separate colors to show their general biological relevance in metabolism, environmental information processing, cellular processes, the organismal system, and human disease. Interestingly, our analysis retrieved most of the pathways (both in MDD and BD) as part of the environmental information processing clan. This is an intriguing finding, given that both MDD and bipolar disorders are the results of environmental insults and are mediated through epigenetic modifiers in the brain [89]. It is equally interesting to highlight that the maximum number of targeted genes is also part of the environmental information processing category. This gives an idea of how peripherally screened miRNAs from MDD and BD patients and their corresponding biological axis in the brain can be epigenetically involved in disease pathogenesis and progression. From the bar plots, we can also notice that PI3-AKT signaling had the maximum number of genes (30) targeted by miRNAs in MDD. Earlier reports from both preclinical and clinical models have suggested the cortico-limbic deficits of PI3-AKT signaling in affective disorders, especially in MDD [90,91]. In BD, we can see the maximum number of target genes (22) as part of the MAPK signaling pathway. Interestingly, the two most common bipolar treatments (e.g., valproic acid and lithium) target MAPK signaling and regulate MAP kinase activity. Mechanistically, valproic acid helps activate the MAPK pathway in the brain [92]. Thus, the biological role of miRNAs in the BD brains relates to their peripheral counterpart, as we found them to be altered in blood circulation. Part of our analysis was also dedicated to mapping the target genes and how they are cross connected with various pathways. In Figure 2A,B, we have presented two Sankey plots showing the connectedness of individual genes (targeted by miRNAs) with shared and/or unique pathways in MDD and BD, respectively. In preparing the graphs, we limited the number of genes to not more than 25 from each pathway to enhance the clarity of the plots. A bubble diagram has also been appended to show the gene counts for respective pathways (Figure 2C). Additionally, we wanted to understand the singularity and relatedness of the two disorders by comparing the targeted pathway they may share. We found that in the BD group a lesser number of genes are targeted by the miRNAs for some of the pathways, including Rap1, Relaxin, actin cytoskeleton, TNF signaling, synaptic vesicle, T-cell receptor signaling, dopaminergic synapse, and calcium signaling compared to the MDD group. However, we want to caution that this does not mean those pathways are not affected in BD. It could be due to the target prediction algorithm and their subsequent functional clustering, which finally adopted fewer pathways associated with genes in the BD group compared to the MDD group. On the other hand, besides PI3-AKT signaling, a sizable gene enrichment can be seen for MAPK and Ras signaling pathways in both the MDD and BD groups. Finally, in Figure 3A,B, we presented the miRNA-target gene network based on a prediction scheme. The networks from the MDD and BD groups were prepared to highlight the degree of connectedness between the miRNAs and their predicted targets. The two networks were filtered to include target genes from the brain database. To increase the strength of the edges (connections) between the nodes, we applied a degree threshold power of 2.0 in the degree filter parameter and plotted the connections by applying the edge bundling feature. Due to the higher degree threshold, the network only included the miRNAs with the strongest connection to their target genes. The network maps highlight the density of the connections, which is more prominent in the MDD group than in the BD group. This could be due to the smaller number of miRNAs in our literature review based on BD. Altogether, our meta-analysis based on the circulating miRNAs from MDD and BD patients reverberate their biological importance in affecting various brain functions under pathological conditions. 

## 12. Conclusions

The biggest challenge of using miRNAs as biomarkers in neuropsychiatry is associated with genetic heterogeneity and extrinsic factors, such as medication, nutrition, or exposure to specific environmental conditions. In some instances, a single miRNA could provide a correlation with disease development and progression. However, in other instances, a combined panel of miRNAs could provide a more effective prognosis of the disease state. 

However, it remains a critical issue to choose the sources for the reliable discovery and reproducible detection of miRNAs as a biomarker in the peripheral circulation. One of the major concerns is the heterogeneity of the sources contributing to peripheral circulation [93]. Thus far, blood-based serum or plasma remains the preferred source of biomarker discovery and analysis. The same remains true for circulatory miRNA studies in many other clinical settings such as oncology, cardiac, pulmonary, and many more [94]. However, the CSF-based analysis of circulatory miRNAs has proven more encouraging in neuropsychiatric conditions due to its higher specificity and sensitivity to the disease conditions. The primary drawback associated with CSF-based studies is the highly invasive nature of the procedure to collect samples. Nevertheless, other factors associated with CSF-based circulatory miRNA study may prove advantageous, especially for CNS-based disorders and neuropsychiatric conditions. An additional factor in the successful discovery and validation of circulating miRNA as a biomarker is the development of a highly sensitive detection assay. This is critical due to the invasive nature of the peripheral blood collection process, which limits the amount of blood drawn from the patients, and the low abundance of miRNA expression in circulating blood. Therefore, applying highly sensitive miRNA detection assay methods such as next-generation sequencing is strongly recommended, and helps analyze thousands of miRNA expressions in parallel [95]. The costs of such high-throughput experiments may impose a limitation. On the contrary, that will also authenticate the results with the detection of a panel of miRNAs while reducing the false positive outcomes [96,97]. 

It is interesting to highlight the ability of miRNAs to cross the blood–brain barrier and reach systemic flow. However, several factors can diminish the level of miRNAs in the peripheral circulation and may significantly compromise their sensitivity as a circulating biomarker. It has recently been suggested that the actively secreted miRNAs enclosed in exosomes can cross the blood–brain barrier (BBB) and are well protected from degradation [98]. However, it is not very clear how the efflux of exosome happens from brain to circulation and many hypotheses have been put forward to understand the mechanisms [99]. The latest research also highlighted that exosomal miRNAs could be processed by the same machinery used in miRNA biogenesis and thus have widespread consequences within the cell by inhibiting the expression of target protein-coding genes [66]. Evidence shows that exosomal miRNAs are excreted physiologically in response to stress and can be ideally used as potential biomarkers. Due to their biogenesis from cellular endocytosis, exosomes contain specific protein markers on their surfaces and often represent their tissue of origin. With this, the neuron-derived exosomal fraction found in peripheral circulation can be selectively immuno-enriched and used in downstream analysis for the successful and reliable detection of brain-derived miRNAs in circulation. Thus, exosomal miRNA cargo originated in the brain can make a significant difference in the peripheral circulation, and boosts confidence for their increased potentiality as a robust molecular biomarker in the diagnosis of most common neuropsychiatric conditions such as MDD, BD, and suicidality. 

Finally, to ascertain the diagnostic value, a more heuristic approach is needed. An association of miRNAs needs to be established with various endophenotypes, given the heterogeneity associated with depression and other psychiatric disorders. Some recent studies suggested that miRNAs play a role in causing susceptibility to developing depression associated with early life trauma [100,101,102]. In this connection, it is highly likely that identifying unique miRNAs may serve as a potential source of screening and they could be used as predictive biomarkers for the early detection of the severity of depression and the treatment response.

Major depression is an episodic disorder with future relapses and complex clinical presentation, including the stratification of different subtypes (melancholic vs. atypical) and responder and non-responder groups [103]. Therefore, identifying objective biomarkers such as circulating miRNAs for improving diagnostic accuracy is critical for designing an effective framework of therapeutic intervention. However, at this point we are yet to distinguish such changes in miRNA expression from the peripheral circulation of MDD patients who are presented with syndrome stratifications. Future research is required to develop more advanced diagnostic panels of miRNAs that can align well with symptomatic peculiarities. Similarly, BD is a complicated disorder. Compared to the general population, BD patients are approximately more than 20 times more likely to die by suicide [104], and the pathogenesis of BD is still not clearly understood. The findings pointed out in this review suggest that circulating miRNAs could serve as biomarkers for BD and can be linked to novel treatment approaches and the prediction of the onset of symptoms. However, compared to MDD, there are fewer studies on BD. The same is true for suicidal behavior. Most often, suicidality is considered endophenotypic of various neuropsychiatric conditions and partly results from behavioral traits, including borderline personality disorder, impulsivity, and adjustment disorder [105]. Such traits are the mediators of suicide risk but are equally influenced by the patient’s clinical state, including how they cope with life stress and alcohol and substance abuse [106,107]. It is believed that under every single condition the miRNA response should be different and can distinctly mark the onset and progression of phenotypic changes that collectively lead to increased risk of suicide [80,108]. Thus, miRNAs can provide a wide spectrum of diagnostic windows as well as predictive biomarkers for the early detection of the risks associated with suicidality. Nonetheless, the predictive values of miRNAs as circulating biomarkers could be further rationalized in future diagnosis and treatment response with careful longitudinal study design that may help to determine subjective changes based on the prognosis of the disease and treatment regime. 

## Figures and Tables

**Table 1:** miRNA expression changes in major depressive disorder, bipolar disorder, and suicidal behavior.

Samples	MiRNA Expression Changes	Reference
Major Depressive Disorder		
Peripheral blood	let-7d-5p, miR-1915-3p, miR-29c-5p, let-7f-5p, miR-330-3p, miR-425-3p, miR-24-3p, let-7a-5p, miR-199a-5p, miR-345-5p	[41]
Blood mononuclear cells	miR-26b, miR-29b, miR-146b, miR-1244, miR- 4485, miR-1972, miR-4498, miR-4743, miR-874, miR-338	[42]
Plasma	miR-320a, miR-451a, miR-17-5p, miR-223-3p, miR-25-3p, miR-126-3p, miR-16-5p, miR-93-5p	[43]
Serum	miR-124-3p	[7]
Blood mononuclear cells	miR-124	[44]
Serum	miR-221-3p	[45]
Cerebrospinal fluid and serum	miR-125a-5p, let-7d-3p, miR-30a-5p, miR-34a-5p, miR-221-3p, miR-29b-3p, miR-10a-5p, miR-375, miR-155–5p, miR-33a-5p, miR-139–5p, miR-451a, miR-15b-5p miR-106b-5p, miR-590–5p, miR-185–5p	[46]
Cerebrospinal fluid and blood	miR-16	[47]
Peripheral blood leukocyte	miR-34b-5p and miR-34c-5p	[48]
Serum	miR-9	[49]
Plasma	miR-483-5p, let-7f-5p, let-7e-5p, miR-122-5p, miR-125a-5p, miR-150-5p, miR-139-3p, miR-193a-5p, miR-125b-5p, miR-197-3p, miR-483-3p	[50]
Blood mononuclear cells	let-7e, miR-21-5p miR-145, miR-223, miR-146a, and miR-155	[51]
Serum	miR-199b-5p, miR-215-5p, miR-200a-3p, miR-143-3p	[52]
Plasma	let-7b-5p	[53]
Peripheral blood	let-7b, let-7c	[54]
Plasma	miR-134	[55]
Peripheral blood	miR-144-3p	[56]
Peripheral blood	miR-130, miR-505, miR-29b-2, miR-26b, miR-22, miR-26a, miR-664, miR-494, let-7d, let-7g, let-7e, miR-34c-5p, let-7f, miR-629, miR-106, miR-103, miR-191, miR-128, miR-502-3p, miR-374b, miR-132, miR-30d, miR-500, miR-770-5p, miR-589, miR-183, miR-574-3p, miR-140-3p, miR-335, miR-361-5p	[57]
Plasma	miR-425-3p, miR-24-3p, miR-503-5p, miR-146a-5p, miR-215-5p, miR-3074-5p, miR-1180-3p, miR-425-5p, miR-324-5p, miR-146b-5p, miR-6750-3p, miR-6511a-3p, miR-361-5p, miR-3173-5p, miR-2110, miR-3605-3p, miR-6881-3p, miR-30e-5p, miR-423-3p, miR-361-3p, miR-3184-5p, miR-636	[58]
Peripheral blood	miR-335	[59]
Plasma	miR-1193, miR-3173-3p, miR-3154, miR-129-5p, miR-3661, miR-1287, miR-532-3p, miR-2278, miR-3150a-3p, miR-3909, miR-151-5p, miR-99b, miR-937, miR-676, miR-223, miR-181b, miR-489, miR-637, miR-608, miR-26a	[60]
Plasma	miR-132, miR-124	[61]
Whole blood	miR-183, miR-212	[62]
Blood exosome	miR-139-5p	[63]
Serum exosome	miR-139-5p	[64]
Serum exosome	let-7e, miR-21-5p, miR-145, miR-146a, miR-155	[65]
Plasma exosome, neuron-derived plasma exosomes	miR-423-3p, miR-191-5p, miR-486-5p, miR-30d-5p, miR-425-5p, miR-25-3p, miR-21-5p, miR-335-5p, miR-126-5p	[66]
**Bipolar Disorder**		
Exosomes from plasma	miR-185-5p, miR-25-3p, miR-92a-3p, miR-376b-3p, let-7i-5p, miR-484, miR-652–3p, miR-142-3p, miR-30b-5p, miR-126–3p, miR-15a-5p, miR-126-5p, miR-301a-3p	[67]
Plasma	miR-21-5p, miR-22-3p, miR-29-c3p, miR-92a-3p, miR-142-3p, miR-1185-2-3p, miR-3135b, miR-3194-5p, miR-4516, miR-6090, miR-6791-5p, miR-6808-5p, miR-7975, miR-7977, miR-133a-3p, miR-188-5p, miR-451a, miR-671-5p, miR-1227-5p, miR-1238-3p, miR-1268b, miR-1281, miR-3620-5p, miR-4433a-5p, miR-5739, miR-6068, miR-6125, miR-6727-5p, miR-6775-5p, miR-6800-3p, miR-6821-5p, miR-7108-5p, miR-8060	[68]
Plasma	miR-132, miR-134, miR-152, miR-607, miR-633, miR-652, miR-15b, miR-155	[69]
Plasma	miR-150-5p, miR-25-3p, miR-451a, miR-144-3p, miR-363-3p, miR-4454+miR-7975, miR-873-3p, miR-548al, miR-598-3p, miR-4443, miR-551a, miR-6721-5p	[70]
Serum	miR-7-5p, miR-23b-3p, miR-142-3p, miR-221-5p, miR-370-3p	[71]
Peripheral whole blood	miR-376a-3p, miR-3680-5p, miR-4253-5p, miR-4482-3p, miR-145-5p	[72]
Blood mononuclear cells	miR-499-5p	[73]
Whole blood	Manic episode: miR-9-5p, miR-29a-3p, miR-106a-5p, miR-106b-5p, miR-107, miR-125a-3p, miR-125b-5p (compared to controls), miR-106a-5p, miR-107 (compared to euthymic episode). euthymic episode: miR-29a-3p, miR-106b-5p, miR-107, miR-125a-3p (compared to controls)	[74]
Blood	miR-15a-5p, miR-17-3p, miR-17-5p, miR-18a-5p, miR-19b-3p, miR-20a-5p, miR-27a-3p, miR-30b-5p, miR-106a-5p, miR-106b-5p, miR-145-5p, miR-148b-3p, miR-210-3p, miR-339-5p, Asenapine: miR-92b-5p, miR-1343-5p. Risperidone: miR-146b-5p, miR-664b-5p, miR-6778-5p	[75]
Plasma	let-7e-5p, miR-125a-5p (compared to controls)	[50]
Plasma	miR-134 (compared to MDD), miR-134 (compared to controls)	[55]
Plasma	miR19b-3p	[76]
Blood mononuclear cells	miR-21-3p, miR-29c-5p, miR-30d-5p, miR-140-3p, miR-330-3p, miR-330-5p, miR-345-5p, miR-378a-5p, miR-720-5p, miR-1973-5p, miR-3158-3p, miR-4521-5p, miR-1915-5p, miR-1972-5p, miR-4440-5p, miR-4793-3p	[41]
Serum	(Correlation with BDNF: miR7-5p, miR221-5p, miR370-3p)	[77]
Whole blood	miR-15b, miR-132, miR-652	[78]
**Suicidal Behavior**		
Plasma	miR-424-5p, miR-378i, miR-6724-5p, and miR-10b-5p	[79]
Biocomputation analysis	miR-27b-3p, miR-124-3p, miR-129-5p, miR-381-3p, miR-3135b, miR-4516 and miR-4286	[80]
Blood mononuclear cells	miR-19a-3p	[81]
Whole blood	miR-3688 and miR-5695	[82]

**Table 2:** Chromosomal Coordinates and Complete Sequence of Major Depressive Disorder and Bipolar Disorder Enriched miRNAs.

Name	Accession	Location	Sequence
Major Depressive Disorder and Bipolar Disorder Enriched miRNAs			
hsa-let-7a-5p	MIMAT0000062	chr22:46,112,752-46,112,773 (+); chr11:122,146,568-122,146,589 (−); chr9:94,175,962-94,175,983 (+)	UGAGGUAGUAGGUUGUAUAGUU
hsa-let-7b-5p	MIMAT0000063	chr22:46,113,691-46,113,712 (+)	UGAGGUAGUAGGUUGUGUGGUU
hsa-let-7d-3p	MIMAT0004484	chr9:94,178,895-94,178,916 (+)	CUAUACGACCUGCUGCCUUUCU
hsa-let-7d-5p	MIMAT0000065	chr9:94,178,841-94,178,862 (+)	AGAGGUAGUAGGUUGCAUAGUU
hsa-let-7f-5p	MIMAT0000067	chr9:94,176,353-94,176,374 (+); chrX:53,557,246-53,557,267 (−)	UGAGGUAGUAGAUUGUAUAGUU
hsa-miR-10a-5p	MIMAT0000253	chr17:48,579,904-48,579,926 (−)	UACCCUGUAGAUCCGAAUUUGUG
hsa-miR-1180-3p	MIMAT0005825	chr17:19,344,513-19,344,534 (−)	UUUCCGGCUCGCGUGGGUGUGU
hsa-miR-1193	MIMAT0015049	chr14:101,030,061-101,030,083 (+)	GGGAUGGUAGACCGGUGACGUGC
hsa-miR-122-5p	MIMAT0000421	chr18:58,451,088-58,451,109 (+)	UGGAGUGUGACAAUGGUGUUUG
hsa-miR-124-3p	MIMAT0000422	chr20:63,178,552-63,178,571 (+); chr8:9,903,401-9,903,420 (−); chr8:64,379,210-64,379,229 (+)	UAAGGCACGCGGUGAAUGCC
hsa-miR-1244	MIMAT0005896	chr12:9,239,472-9,239,497 (−); chr12:12,112,006-12,112,031 (+); chr2:231,713,368-231,713,393 (+); chr5:118,974,640-118,974,665 (+)	AAGUAGUUGGUUUGUAUGAGAUGGUU
hsa-miR-126-3p	MIMAT0000445	chr9:136,670,653-136,670,674 (+)	UCGUACCGUGAGUAAUAAUGCG
hsa-miR-126-5p	MIMAT0000444	chr9:136,670,616-136,670,636 (+)	CAUUAUUACUUUUGGUACGCG
hsa-miR-1275	MIMAT0005929	chr6:34,000,018-34,000,034 (−)	GUGGGGGAGAGGCUGUC
hsa-miR-129-5p	MIMAT0000242	chr7:128,207,876-128,207,896 (+); chr11:43,581,408-43,581,428 (+)	CUUUUUGCGGUCUGGGCUUGC
hsa-miR-139-3p	MIMAT0004552	chr11:72,615,066-72,615,088 (−)	UGGAGACGCGGCCCUGUUGGAGU
hsa-miR-139-5p	MIMAT0000250	chr11:72,615,102-72,615,124 (−)	UCUACAGUGCACGUGUCUCCAGU
hsa-miR-143-3p	MIMAT0000435	chr5:149,428,978-149,428,998 (+)	UGAGAUGAAGCACUGUAGCUC
hsa-miR-146a-5p	MIMAT0000449	chr5:160,485,372-160,485,393 (+)	UGAGAACUGAAUUCCAUGGGUU
hsa-miR-15b-5p	MIMAT0000417	chr3:160,404,607-160,404,628 (+)	UAGCAGCACAUCAUGGUUUACA
hsa-miR-16-5p	MIMAT0000069	chr3:160,404,754-160,404,775 (+); chr13:50,049,027-50,049,048 (−)	UAGCAGCACGUAAAUAUUGGCG
hsa-miR-191-5p	MIMAT0000440	chr3:49,020,672-49,020,694 (−)	CAACGGAAUCCCAAAAGCAGCUG
hsa-miR-1915-3p	MIMAT0007892	chr10:21,496,576-21,496,595 (−)	CCCCAGGGCGACGCGGCGGG
hsa-miR-193a-5p	MIMAT0004614	chr17:31,560,016-31,560,037 (+)	UGGGUCUUUGCGGGCGAGAUGA
hsa-miR-197-3p	MIMAT0000227	chr1:109,598,940-109,598,961 (+)	UUCACCACCUUCUCCACCCAGC
hsa-miR-1972	MIMAT0009447	chr16:15,010,329-15,010,350 (−); chr16:70,030,393-70,030,414 (+)	UCAGGCCAGGCACAGUGGCUCA
hsa-miR-199a-5p	MIMAT0000231	chr19:10,817,469-10,817,491 (−); chr1:172,144,592-172,144,614 (−)	CCCAGUGUUCAGACUACCUGUUC
hsa-miR-199b-5p	MIMAT0000263	chr9:128,244,783-128,244,805 (−)	CCCAGUGUUUAGACUAUCUGUUC
hsa-miR-200a-3p	MIMAT0000682	chr1:1,167,916-1,167,937 (+)	UAACACUGUCUGGUAACGAUGU
hsa-miR-2110	MIMAT0010133	chr10:114,174,151-114,174,172 (−)	UUGGGGAAACGGCCGCUGAGUG
hsa-miR-215-5p	MIMAT0000272	chr1:220,117,916-220,117,936 (−)	AUGACCUAUGAAUUGACAGAC
hsa-miR-221-3p	MIMAT0000278	chrX:45,746,180-45,746,202 (−)	AGCUACAUUGUCUGCUGGGUUUC
hsa-miR-223-3p	MIMAT0000280	chrX:66,018,937-66,018,958 (+)	UGUCAGUUUGUCAAAUACCCCA
hsa-miR-2278	MIMAT0011778	chr9:94,809,977-94,809,998 (+)	GAGAGCAGUGUGUGUUGCCUGG
hsa-miR-24-3p	MIMAT0000080	chr9:95,086,064-95,086,085 (+); chr19:13,836,289-13,836,310 (−)	UGGCUCAGUUCAGCAGGAACAG
hsa-miR-29b-3p	MIMAT0000100	chr7:130,877,467-130,877,489 (−); chr1:207,802,450-207,802,472 (−)	UAGCACCAUUUGAAAUCAGUGUU
hsa-miR-3074-5p	MIMAT0019208	chr9:95,086,063-95,086,083 (−)	GUUCCUGCUGAACUGAGCCAG
hsa-miR-30a-5p	MIMAT0000087	chr6:71,403,595-71,403,616 (−)	UGUAAACAUCCUCGACUGGAAG
hsa-miR-30e-3p	MIMAT0000693	chr1:40,754,413-40,754,434 (+)	CUUUCAGUCGGAUGUUUACAGC
hsa-miR-30e-5p	MIMAT0000692	chr1:40,754,371-40,754,392 (+)	UGUAAACAUCCUUGACUGGAAG
hsa-miR-3150a-3p	MIMAT0015023	chr8:95,072,962-95,072,983 (+)	CUGGGGAGAUCCUCGAGGUUGG
hsa-miR-3154	MIMAT0015028	chr9:128,244,956-128,244,977 (−)	CAGAAGGGGAGUUGGGAGCAGA
hsa-miR-3173-3p	MIMAT0015048	chr14:95,137,923-95,137,944 (−)	AAAGGAGGAAAUAGGCAGGCCA
hsa-miR-3173-5p	MIMAT0019214	chr14:95,137,961-95,137,982 (−)	UGCCCUGCCUGUUUUCUCCUUU
hsa-miR-3184-5p	MIMAT0015064	chr17:30,117,128-30,117,151 (−)	UGAGGGGCCUCAGACCGAGCUUUU
hsa-miR-320a	MIMAT0000510	chr8:22,244,975-22,244,996 (−)	AAAAGCUGGGUUGAGAGGGCGA
hsa-miR-324-5p	MIMAT0000761	chr17:7,223,342-7,223,364 (−)	CGCAUCCCCUAGGGCAUUGGUGU
hsa-miR-335-5p	MIMAT0000765	chr7:130,496,126-130,496,148 (+)	UCAAGAGCAAUAACGAAAAAUGU
hsa-miR-33a-5p	MIMAT0000091	chr22:41,900,949-41,900,969 (+)	GUGCAUUGUAGUUGCAUUGCA
hsa-miR-34a-5p	MIMAT0000255	chr1:9,151,735-9,151,756 (−)	UGGCAGUGUCUUAGCUGGUUGU
hsa-miR-34b-5p	MIMAT0000685	chr11:111,512,950-111,512,972 (+)	UAGGCAGUGUCAUUAGCUGAUUG
hsa-miR-34c-5p	MIMAT0000686	chr11:111,513,451-111,513,473 (+)	AGGCAGUGUAGUUAGCUGAUUGC
hsa-miR-3605-3p	MIMAT0017982	chr1:33,332,405-33,332,427 (−)	CCUCCGUGUUACCUGUCCUCUAG
hsa-miR-361-3p	MIMAT0004682	chrX:85,903,641-85,903,663 (−)	UCCCCCAGGUGUGAUUCUGAUUU
hsa-miR-361-5p	MIMAT0000703	chrX:85,903,681-85,903,702 (−)	UUAUCAGAAUCUCCAGGGGUAC
hsa-miR-3611	MIMAT0017988	chr10:35,079,609-35,079,629 (−)	UUGUGAAGAAAGAAAUUCUUA
hsa-miR-3661	MIMAT0018082	chr5:134,225,777-134,225,798 (+)	UGACCUGGGACUCGGACAGCUG
hsa-miR-375	MIMAT0000728	chr2:219,001,648-219,001,669 (−)	UUUGUUCGUUCGGCUCGCGUGA
hsa-miR-3909	MIMAT0018183	chr22:35,335,710-35,335,731 (+)	UGUCCUCUAGGGCCUGCAGUCU
hsa-miR-3944-5p	MIMAT0019231	chr10:133,371,621-133,371,641 (−)	UGUGCAGCAGGCCAACCGAGA
hsa-miR-423-3p	MIMAT0001340	chr17:30,117,131-30,117,153 (+)	AGCUCGGUCUGAGGCCCCUCAGU
hsa-miR-425-3p	MIMAT0001343	chr3:49,020,159-49,020,180 (−)	AUCGGGAAUGUCGUGUCCGCCC
hsa-miR-425-5p	MIMAT0003393	chr3:49,020,199-49,020,221 (−)	AAUGACACGAUCACUCCCGUUGA
hsa-miR-4498	MIMAT0019033	chr12:120,155,473-120,155,494 (−)	UGGGCUGGCAGGGCAAGUGCUG
hsa-miR-4532	MIMAT0019071	chr20:57,895,399-57,895,415 (+)	CCCCGGGGAGCCCGGCG
hsa-miR-483-3p	MIMAT0002173	chr11:2,134,142-2,134,162 (−)	UCACUCCUCUCCUCCCGUCUU
hsa-miR-483-5p	MIMAT0004761	chr11:2,134,181-2,134,202 (−)	AAGACGGGAGGAAAGAAGGGAG
hsa-miR-486-5p	MIMAT0002177	chr8:41,660,444-41,660,465 (+); chr8:41,660,484-41,660,505 (−)	UCCUGUACUGAGCUGCCCCGAG
hsa-miR-502-3p	MIMAT0004775	chrX:50,014,649-50,014,670 (+)	AAUGCACCUGGGCAAGGAUUCA
hsa-miR-503-5p	MIMAT0002874	chrX:134,546,371-134,546,393 (−)	UAGCAGCGGGAACAGUUCUGCAG
hsa-miR-532-3p	MIMAT0004780	chrX:50,003,204-50,003,225 (+)	CCUCCCACACCCAAGGCUUGCA
hsa-miR-574-3p	MIMAT0003239	chr4:38,868,092-38,868,113 (+)	CACGCUCAUGCACACACCCACA
hsa-miR-608	MIMAT0003276	chr10:100,975,000-100,975,024 (+)	AGGGGUGGUGUUGGGACAGCUCCGU
hsa-miR-636	MIMAT0003306	chr17:76,736,466-76,736,488 (−)	UGUGCUUGCUCGUCCCGCCCGCA
hsa-miR-637	MIMAT0003307	chr19:3,961,429-3,961,452 (−)	ACUGGGGGCUUUCGGGCUCUGCGU
hsa-miR-6511a-3p	MIMAT0025479	chr16:14,925,980-14,926,001 (+); chr16:16,324,631-16,324,652 (+); chr16:16,368,919-16,368,940 (+); chr16:18,344,015-18,344,036 (−)	CCUCACCAUCCCUUCUGCCUGC
hsa-miR-6750-3p	MIMAT0027401	chr11:64,898,363-64,898,383 (−)	GAACUCACCCUCUGCUCCCAG
hsa-miR-6881-3p	MIMAT0027663	chr15:74,411,361-74,411,382 (−)	AUCCUCUUUCGUCCUUCCCACU
hsa-miR-770-5p	MIMAT0003948	chr14:100,852,409-100,852,431 (+)	UCCAGUACCACGUGUCAGGGCCA
hsa-miR-93-5p	MIMAT0000093	chr7:100,093,815-100,093,837 (−)	CAAAGUGCUGUUCGUGCAGGUAG
**Bipolar Disorder Enriched miRNAs**			
hsa-let-7i-5p	MIMAT0000415	chr12:62,603,691-62,603,712 (+)	UGAGGUAGUAGUUUGUGCUGUU
hsa-miR-106a-5p	MIMAT0000103	chrX:134,170,244-134,170,266 (−)	AAAAGUGCUUACAGUGCAGGUAG
hsa-miR-107	MIMAT0000104	chr10:89,592,756-89,592,778 (−)	AGCAGCAUUGUACAGGGCUAUCA
hsa-miR-1185-2-3p	MIMAT0022713	chr14:101,044,250-101,044,271 (+)	AUAUACAGGGGGAGACUCUCAU
hsa-miR-1227-5p	MIMAT0022941	chr19:2,234,133-2,234,149 (−)	GUGGGGCCAGGCGGUGG
hsa-miR-1238-3p	MIMAT0005593	chr19:10,552,183-10,552,202 (+)	CUUCCUCGUCUGUCUGCCCC
hsa-miR-125a-3p	MIMAT0004602	chr19:51,693,306-51,693,327 (+)	ACAGGUGAGGUUCUUGGGAGCC
hsa-miR-1268b	MIMAT0018925	chr17:80,098,831-80,098,850 (+)	CGGGCGUGGUGGUGGGGGUG
hsa-miR-1281	MIMAT0005939	chr22:41,092,545-41,092,561 (+)	UCGCCUCCUCCUCUCCC
hsa-miR-133a-3p	MIMAT0000427	chr18:21,825,712-21,825,733 (−); chr20:62,564,970-62,564,991 (+)	UUUGGUCCCCUUCAACCAGCUG
hsa-miR-1343-5p	MIMAT0027038	chr11:34,941,851-34,941,872 (+)	UGGGGAGCGGCCCCCGGGUGGG
hsa-miR-142-3p	MIMAT0000434	chr17:58,331,245-58,331,267 (−)	UGUAGUGUUUCCUACUUUAUGGA
hsa-miR-145-5p	MIMAT0000437	chr5:149,430,661-149,430,683 (+)	GUCCAGUUUUCCCAGGAAUCCCU
hsa-miR-15a-5p	MIMAT0000068	chr13:50,049,167-50,049,188 (−)	UAGCAGCACAUAAUGGUUUGUG
hsa-miR-17-3p	MIMAT0000071	chr13:91,350,655-91,350,676 (+)	ACUGCAGUGAAGGCACUUGUAG
hsa-miR-188-5p	MIMAT0000457	chrX:50,003,517-50,003,537 (+)	CAUCCCUUGCAUGGUGGAGGG
hsa-miR-18a-5p	MIMAT0000072	chr13:91,350,756-91,350,778 (+)	UAAGGUGCAUCUAGUGCAGAUAG
hsa-miR-1915-5p	MIMAT0007891	chr10:21,496,609-21,496,630 (−)	ACCUUGCCUUGCUGCCCGGGCC
hsa-miR-19b-3p	MIMAT0000074	chr13:91,351,245-91,351,267 (+); chrX:134,169,683-134,169,705 (−)	UGUGCAAAUCCAUGCAAAACUGA
hsa-miR-20a-5p	MIMAT0000075	chr13:91,351,072-91,351,094 (+)	UAAAGUGCUUAUAGUGCAGGUAG
hsa-miR-21-3p	MIMAT0004494	chr17:59,841,311-59,841,331 (+)	CAACACCAGUCGAUGGGCUGU
hsa-miR-210-3p	MIMAT0000267	chr11:568,112-568,133 (−)	CUGUGCGUGUGACAGCGGCUGA
hsa-miR-22-3p	MIMAT0000077	chr17:1,713,914-1,713,935 (−)	AAGCUGCCAGUUGAAGAACUGU
hsa-miR-221-5p	MIMAT0004568	chrX:45,746,221-45,746,242 (−)	ACCUGGCAUACAAUGUAGAUUU
hsa-miR-23b-3p	MIMAT0000418	chr9:95,085,265-95,085,285 (+)	AUCACAUUGCCAGGGAUUACC
hsa-miR-27a-3p	MIMAT0000084	chr19:13,836,447-13,836,467 (−)	UUCACAGUGGCUAAGUUCCGC
hsa-miR-29a-3p	MIMAT0000086	chr7:130,876,748-130,876,769 (−)	UAGCACCAUCUGAAAUCGGUUA
hsa-miR-301a-3p	MIMAT0000688	chr17:59,151,149-59,151,171 (−)	CAGUGCAAUAGUAUUGUCAAAGC
hsa-miR-30b-5p	MIMAT0000420	chr8:134,800,570-134,800,591 (−)	UGUAAACAUCCUACACUCAGCU
hsa-miR-3135b	MIMAT0018985	chr6:32,749,952-32,749,973 (−)	GGCUGGAGCGAGUGCAGUGGUG
hsa-miR-3194-5p	MIMAT0015078	chr20:51,452,948-51,452,968 (−)	GGCCAGCCACCAGGAGGGCUG
hsa-miR-330-5p	MIMAT0004693	chr19:45,639,049-45,639,070 (−)	UCUCUGGGCCUGUGUCUUAGGC
hsa-miR-339-5p	MIMAT0000764	chr7:1,022,990-1,023,012 (−)	UCCCUGUCCUCCAGGAGCUCACG
hsa-miR-3620-5p	MIMAT0022967	chr1:228,097,285-228,097,306 (+)	GUGGGCUGGGCUGGGCUGGGCC
hsa-miR-363-3p	MIMAT0000707	chrX:134,169,382-134,169,403 (−)	AAUUGCACGGUAUCCAUCUGUA
hsa-miR-3680-5p	MIMAT0018106	chr16:29,599,231-29,599,252 (−); chr16:21,506,101-21,506,122 (−)	GACUCACUCACAGGAUUGUGCA
hsa-miR-370-3p	MIMAT0000722	chr14:100,911,186-100,911,207 (+)	GCCUGCUGGGGUGGAACCUGGU
hsa-miR-376a-3p	MIMAT0000729	chr14:101,040,825-101,040,845 (+); chr14:101,040,118-101,040,138 (+)	AUCAUAGAGGAAAAUCCACGU
hsa-miR-376b-3p	MIMAT0002172	chr14:101,040,497-101,040,518 (+)	AUCAUAGAGGAAAAUCCAUGUU
hsa-miR-378a-5p	MIMAT0000731	chr5:149,732,829-149,732,850 (+)	CUCCUGACUCCAGGUCCUGUGU
hsa-miR-4433a-5p	MIMAT0020956	chr2:64,340,770-64,340,790 (+)	CGUCCCACCCCCCACUCCUGU
hsa-miR-4443	MIMAT0018961	chr3:48,196,572-48,196,588 (+)	UUGGAGGCGUGGGUUUU
hsa-miR-4454	MIMAT0018976	chr4:163,093,607-163,093,626 (−)	GGAUCCGAGUCACGGCACCA
hsa-miR-4482-3p	MIMAT0020958	chr10:104,268,341-104,268,362 (−)	UUUCUAUUUCUCAGUGGGGCUC
hsa-miR-4793-3p	MIMAT0019966	chr3:48,644,201-48,644,223 (−)	UCUGCACUGUGAGUUGGCUGGCU
hsa-miR-484	MIMAT0002174	chr16:15,643,301-15,643,322 (+)	UCAGGCUCAGUCCCCUCCCGAU
hsa-miR-548al	MIMAT0019024	chr11:74,399,301-74,399,322 (+)	AACGGCAAUGACUUUUGUACCA
hsa-miR-551a	MIMAT0003214	chr1:3,560,710-3,560,730 (−)	GCGACCCACUCUUGGUUUCCA
hsa-miR-5739	MIMAT0023116	chr22:28,459,925-28,459,944 (+)	GCGGAGAGAGAAUGGGGAGC
hsa-miR-598-3p	MIMAT0003266	chr8:11,035,221-11,035,242 (−)	UACGUCAUCGUUGUCAUCGUCA
hsa-miR-6068	MIMAT0023693	chr1:63,326,964-63,326,984 (−)	CCUGCGAGUCUCCGGCGGUGG
hsa-miR-607	MIMAT0003275	chr10:96,828,684-96,828,704 (−)	GUUCAAAUCCAGAUCUAUAAC
hsa-miR-6090	MIMAT0023715	chr11:128,522,430-128,522,448 (+)	GGGGAGCGAGGGGCGGGGC
hsa-miR-6125	MIMAT0024598	chr12:62,260,418-62,260,437 (+)	GCGGAAGGCGGAGCGGCGGA
hsa-miR-633	MIMAT0003303	chr17:62,944,275-62,944,297 (+)	CUAAUAGUAUCUACCACAAUAAA
hsa-miR-664b-5p	MIMAT0022271	chrX:154,768,596-154,768,619 (+)	UGGGCUAAGGGAGAUGAUUGGGUA
hsa-miR-671-5p	MIMAT0003880	chr7:151,238,449-151,238,471 (+)	AGGAAGCCCUGGAGGGGCUGGAG
hsa-miR-6721-5p	MIMAT0025852	chr6:32,170,084-32,170,106 (−)	UGGGCAGGGGCUUAUUGUAGGAG
hsa-miR-6727-5p	MIMAT0027355	chr1:1,312,539-1,312,561 (−)	CUCGGGGCAGGCGGCUGGGAGCG
hsa-miR-6775-5p	MIMAT0027450	chr16:87,834,631-87,834,655 (−)	UCGGGGCAUGGGGGAGGGAGGCUGG
hsa-miR-6778-5p	MIMAT0027456	chr17:18,340,860-18,340,881 (−)	AGUGGGAGGACAGGAGGCAGGU
hsa-miR-6791-5p	MIMAT0027482	chr19:6,736,752-6,736,773 (−)	CCCCUGGGGCUGGGCAGGCGGA
hsa-miR-6800-3p	MIMAT0027501	chr19:49,832,076-49,832,096 (+)	CACCUCUCCUGGCAUCGCCCC
hsa-miR-6808-5p	MIMAT0027516	chr1:1,339,682-1,339,703 (−)	CAGGCAGGGAGGUGGGACCAUG
hsa-miR-6821-5p	MIMAT0027542	chr22:49,962,866-49,962,888 (+)	GUGCGUGGUGGCUCGAGGCGGGG
hsa-miR-7-5p	MIMAT0000252	chr9:83,969,812-83,969,834 (−); chr19:4,770,700-4,770,722 (+); chr15:88,611,856-88,611,878 (+)	UGGAAGACUAGUGAUUUUGUUGU
hsa-miR-7108-5p	MIMAT0028113	chr19:2,434,980-2,435,000 (−)	GUGUGGCCGGCAGGCGGGUGG
hsa-miR-7975	MIMAT0031178	chr19:55,123,225-55,123,242 (−)	AUCCUAGUCACGGCACCA
hsa-miR-7977	MIMAT0031180	chr3:176,515,103-176,515,120 (+)	UUCCCAGCCAACGCACCA
hsa-miR-8060	MIMAT0030987	chr3:96,360,006-96,360,029 (+)	CCAUGAAGCAGUGGGUAGGAGGAC
hsa-miR-873-3p	MIMAT0022717	chr9:28,888,889-28,888,910 (−)	GGAGACUGAUGAGUUCCCGGGA
hsa-miR-9-5p	MIMAT0000441	chr5:88,666,902-88,666,924 (−); chr1:156,420,392-156,420,414 (−); chr15:89,368,032-89,368,054 (+)	UCUUUGGUUAUCUAGCUGUAUGA
hsa-miR-92a-3p	MIMAT0000092	chr13:91,351,361-91,351,382 (+); chrX:134,169,544-134,169,565 (−)	UAUUGCACUUGUCCCGGCCUGU
hsa-miR-92b-5p	MIMAT0004792	chr1:155,195,196-155,195,217 (+)	AGGGACGGGACGCGGUGCAGUG

## Data Availability

The data are contained within the article.
